# Some Unfounded Statements at Liverpool

**Published:** 1918-02-16

**Authors:** 


### THE STATUS OF NURSES.

### Some Unfounded Statements at Liverpool.

**[The "Times" of the 8th inst. published a letter headed "The Status of Nurses," signed by the Hon. Sir Arthur Stanley, G.B.E., M.P., Chairman of the College of Nursing. We have received a copy of the whole letter which Sir Arthur Stanley sent to "The Times," and in view of the extraordinary statements made at the R.B.N.A. meeting at Liverpool on the 8th inst., of which a report will be found on page 424, we are publishing this letter in full, marking with a black line at the side the two paragraphs which were omitted by the Editor of "The Times." These paragraphs have for the nursing world special interest and great importance, as our readers in the hospital world will recognise. The final paragraph, it will be seen, proves beyond dispute that some of Mr. Paterson's statements in regard to the College are devoid of foundation in fact.—Ed. "The Hospital."]**

